# Supplementary material for: Hospital-Based Genomic Surveillance of Klebsiella pneumoniae: Trends in Resistance and Infection
Source: Biology (Basel). 2025 Dec 16;14(12):1795. doi: 10.3390/biology14121795 (PMC12731027; doi:10.3390/biology14121795)

Hospital-Based Genomic Surveillance of *Klebsiella pneumoniae* : Trends in Resistance and Infection

Localization in Phylogenetic Tree (Figure 1)	Gender	Age Category Young-age (18-44) Middle-age (45-64) Old-age (over 65)	Age (years old)	*Klebsiella* sp isolates presence	Sample Origin	Comorbidities declared	Bacterial infections declared (last 12 months)	Number hospitalizations (last 12 months)
Cluster 1	Female	Old-age	73	Colonization	Urinary Tract	Yes	No	0
Cluster 1	Female	Old-age	81	Colonization	Urinary Tract	Yes	No	0
Cluster 1	Male	Middle-age	62	Colonization	Urinary Tract	Yes	Yes	1
Cluster 2	Female	Middle-age	56	Infection	Central Venous Catheter	Yes	No	1
Cluster 2	Male	Old-age	68	Unknown	Unknown	Yes	No	0
Cluster 2	Male	Old-age	66	Colonization	Urinary Tract	Yes	Yes	0
Cluster 2	Male	Old-age	76	Infection	Urinary Tract	Yes	Yes	2
Cluster 2	Male	Old-age	82	Infection	Urinary Tract	Yes	Yes	0
Cluster 2	Male	Middle-age	62	Infection	Urinary Tract	No	Yes	3
Cluster 3	Female	Old-age	68	Colonization	Urinary Tract	Yes	No	1
Cluster 3	Female	Old-age	74	Colonization	Other	Yes	No	2
Cluster 4	Female	Middle-age	54	Colonization	Other	Yes	No	1
Cluster 4	Male	Old-age	65	Colonization	Other	Yes	No	0
Cluster 4	Female	Old-age	73	Colonization	Other	Yes	No	0
Cluster 4	Male	Old-age	81	Colonization	Other	Yes	No	0
Cluster 4	Female	Young-age	23	Colonization	Other	No	Yes	2
Cluster 4	Male	Old-age	67	Colonization	Other	Yes	No	0
Cluster 4	Female	Middle-age	64	Infection	Urinary Tract	Yes	No	1
Cluster 4	Male	Old-age	65	Colonization	Urinary Tract	Yes	No	3
Cluster 4	Female	Old-age	87	Colonization	Urinary Tract	Yes	Yes	0
Cluster 4	Female	Young-age	34	Infection	Bone	Yes	Yes	4
Cluster 4	Female	Old-age	87	Colonization	Other	Yes	Yes	3
Cluster 4	Female	Old-age	82	Colonization	Urinary Tract	Yes	No	0
Cluster 4	Male	Middle-age	61	Colonization	Other	Yes	Yes	1
Cluster 5	Female	Old-age	83	Infection	Urinary Tract	Yes	Yes	1
Cluster 5	Male	Old-age	79	Infection	Skin/Soft Tissue	Yes	Yes	1
Cluster 6	Male	Middle-age	55	Infection	Respiratory System	No	Yes	2
Cluster 6	Male	Middle-age	56	Infection	Skin/Soft Tissue	Yes	Yes	2
Cluster 7	Male	Old-age	97	Infection	Respiratory System	Yes	No	0
Cluster 7	Female	Old-age	66	Infection	Urinary Tract	No	No	0
Cluster 7	Male	Old-age	80	Colonization	Other	Yes	No	0
Cluster 7	Female	Old-age	76	Infection	Urinary Tract	Yes	Yes	4
Cluster 7	Male	Old-age	77	Colonization	Other	Yes	No	0
Cluster 8	Male	Middle-age	50	Infection	Urinary Tract	Yes	Yes	6
Cluster 8	Male	Old-age	88	Infection	Urinary Tract	Yes	No	0
Cluster 8	Male	Middle-age	58	Colonization	Respiratory System	Yes	No	0
Cluster 8	Female	Old-age	95	Colonization	Other	Yes	Yes	0
Cluster 8	Male	Middle-age	46	Colonization	Other	Yes	No	0
Cluster 8	Male	Old-age	79	Colonization	Urinary Tract	Yes	No	4
Cluster 8	Male	Old-age	85	Colonization	Other	Yes	No	0
Cluster 9	Female	Old-age	86	Infection	Urinary Tract	Yes	Yes	0
Cluster 9	Female	Old-age	65	Infection	Skin/Soft Tissue	No	No	0
Cluster 9	Male	Young-age	39	Colonization	Lymphatic System	No	Yes	1
Cluster 9	Female	Old-age	72	Infection	Urinary Tract	Yes	Yes	2
Cluster 9	Male	Middle-age	57	Infection	Bone	Yes	No	0
Cluster 9	Male	Old-age	65	Colonization	Urinary Tract	Yes	No	0
Cluster 9	Male	Young-age	26	Infection	Abdomen	No	No	0
Cluster 10	Female	Old-age	88	Infection	Urinary Tract	Yes	No	0
Cluster 10	Female	Middle-age	69	Infection	Urinary Tract	Yes	No	0
Cluster 11	Female	Old-age	65	Infection	Urinary Tract	No	No	0
Cluster 11	Female	Old-age	76	Infection	Urinary Tract	Yes	Yes	1
Cluster 11	Male	Middle-age	64	Colonization	Respiratory System	Yes	Yes	5
Cluster 11	Male	Old-age	67	Infection	Urinary Tract	No	Yes	4
Cluster 12	Male	Old-age	74	Infection	Urinary Tract	Yes	Yes	1
Cluster 12	Female	Young-age	34	Colonization	Other	Yes	Yes	4
Cluster 12	Female	Young-age	34	Infection	Bacteremia	Yes	Yes	4
Cluster 12	Male	Old-age	65	Colonization	Other	Yes	No	0
Cluster 12	Male	Old-age	72	Colonization	Other	Yes	No	0
Cluster 12	Female	Middle-age	61	Colonization	Other	Yes	No	0
Cluster 12	Male	Old-age	89	Colonization	Other	Yes	No	0
Cluster 12	Male	Old-age	78	Colonization	Other	Yes	No	0
Cluster 12	Male	Middle-age	56	Colonization	Other	No	No	0
Cluster 12	Female	Old-age	74	Colonization	Other	No	No	0
Cluster 12	Male	Old-age	85	Infection	Urinary Tract	Yes	No	0
Cluster 12	Male	Old-age	81	Colonization	Other	Yes	No	1
Cluster 12	Male	Old-age	81	Colonization	Urinary Tract	Yes	No	1
Cluster 12	Female	Old-age	85	Colonization	Other	Yes	No	1
Cluster 12	Male	Old-age	76	Colonization	Urinary Tract	Yes	Yes	0
Cluster 12	Male	Middle-age	58	Colonization	Other	Yes	No	2
Cluster 12	Female	Young-age	18	Colonization	Urinary Tract	No	Yes	1
Cluster 12	Female	Old-age	72	Colonization	Other	Yes	No	0
Cluster 12	Male	Young-age	22	Infection	Abdomen	No	No	0
Cluster 12	Male	Old-age	71	Infection	Skin/Soft Tissue	Yes	No	0
Cluster 12	Female	Old-age	85	Colonization	Urinary Tract	Yes	No	0
Cluster 12	Female	Old-age	85	Unknown	Unknown	Yes	No	0
Cluster 12	Male	Young-age	22	Infection	Urinary Tract	No	Yes	1
Cluster 12	Female	Old-age	76	Infection	Urinary Tract	Yes	Yes	3
Cluster 12	Male	Old-age	81	Colonization	Other	Yes	Yes	0
Cluster 12	Male	Middle-age	58	Colonization	Other	Yes	No	0
Cluster 12	Female	Old-age	81	Colonization	Other	Yes	No	0
Cluster 12	Male	Middle-age	52	Infection	Abdomen	Yes	No	1
Cluster 12	Female	Old-age	68	Infection	Abdomen	Yes	No	0
Cluster 12	Female	Old-age	67	Infection	Abdomen	No	No	1
Cluster 12	Male	Old-age	71	Infection	Urinary Tract	Yes	No	0
Cluster 12	Female	Old-age	75	Colonization	Other	Yes	No	1
Cluster 12	Male	Old-age	72	Colonization	Urinary Tract	Yes	Yes	0
Cluster 13	Male	Middle-age	50	Infection	Urinary Tract	Yes	No	1
Cluster 13	Female	Middle-age	54	Colonization	Urinary Tract	Yes	Yes	1
Cluster 13	Male	Old-age	73	Infection	Urinary Tract	Yes	No	1
Cluster 13	Male	Old-age	68	Infection	Urinary Tract	Yes	No	0
Cluster 13	Male	Old-age	70	Infection	Urinary Tract	Yes	Yes	1
Cluster 14	Male	Middle-age	47	Infection	Skin/Soft Tissue	Yes	No	0
Cluster 14	Male	Middle-age	64	Infection	Urinary Tract	Yes	Yes	2
Cluster Kka3	Female	Young-age	34	Infection	Bacteremia	Yes	Yes	4
Cluster Kka3	Female	Young-age	34	Infection	Bone	Yes	Yes	4
Cluster Kka3	Female	Old-age	73	Colonization	Other	Yes	No	1
Isolate 1	Male	Old-age	67	Infection	Skin/Soft Tissue	Yes	Yes	1
Isolate 2	Male	Middle-age	64	Infection	Urinary Tract	Yes	Yes	4
Isolate 3	Female	Old-age	71	Colonization	Urinary Tract	Yes	No	0
Isolate 4	Male	Old-age	66	Infection	Urinary Tract	Yes	No	0
Isolate 5	Female	Old-age	78	Infection	Skin/Soft Tissue	Yes	No	1
Isolate 6	Male	Old-age	70	Infection	Abdomen	Yes	No	1
Isolate 7	Male	Middle-age	64	Infection	Abdomen	Yes	No	0
Isolate 8	Female	Old-age	87	Infection	Urinary Tract	Yes	No	2
Isolate 9	Female	Young-age	30	Infection	Abdomen	No	No	0
Isolate 10	Female	Old-age	75	Infection	Urinary Tract	Yes	No	1
Isolate 11	Male	Old-age	82	Infection	Urinary Tract	Yes	No	0
Isolate 12	Male	Middle-age	51	Infection	Abdomen	Yes	No	0
Isolate 13	Female	Young-age	34	Infection	Bone	Yes	Yes	4
Isolate 14	Male	Middle-age	45	Infection	Urinary Tract	No	No	0
Isolate 15	Female	Middle-age	49	Infection	Bacteremia	No	Yes	3
Kvar Isolate 16	Male	Old-age	78	Infection	Respiratory System	Yes	No	0
Kvar Isolate 17	Male	Old-age	83	Infection	Abdomen	Yes	No	0
Kvar Isolate 18	Male	Middle-age	63	Colonization	Other	Yes	No	0
Kvar Isolate 19	Male	Middle-age	60	Colonization	Other	Yes	No	0
Reference genome								

				KLEBORATE	KLEBORATE	KLEBORATE	KLEBORATE	KLEBORATE	KLEBORATE
**Number antibiotics taken in hospital before *Klebsiella* isolation**	**Number of intra-hospital transfers (PCU)**	**In-hospital medical devices**	**Type of *Klebsiella* sp**	**Taxonomy ID (species)**	**QC warnings**	**Virulence score V-score**	**Resistance score R-score**	**Number of resistance classes**	**Number of resistance genes**
1	2	Yes	Nosocomial	Klebsiella pneumoniae	-	0	1	8	8
2	2	Yes	Nosocomial	Klebsiella pneumoniae	-	0	1	8	8
4	2	Yes	Nosocomial	Klebsiella pneumoniae	-	0	1	8	10
2	1	Yes	Nosocomial	Klebsiella pneumoniae	-	1	1	8	12
0	2	Yes	Nosocomial	Klebsiella pneumoniae	-	1	1	8	10
8	3	Yes	Nosocomial	Klebsiella pneumoniae	-	1	1	8	12
1	2	Yes	Nosocomial	Klebsiella pneumoniae	-	1	1	8	12
2	1	Yes	Nosocomial	Klebsiella pneumoniae	-	0	1	8	12
0	1	Yes	Nosocomial	Klebsiella pneumoniae	-	0	1	8	10
1	1	Yes	Nosocomial	Klebsiella pneumoniae	-	0	0	1	1
3	3	Yes	Nosocomial	Klebsiella pneumoniae	-	0	0	1	1
1	1	Yes	Nosocomial	Klebsiella pneumoniae	-	1	2	9	16
0	1	Yes	Nosocomial	Klebsiella pneumoniae	-	1	2	9	16
1	1	Yes	Nosocomial	Klebsiella pneumoniae	-	1	2	9	16
1	1	Yes	Nosocomial	Klebsiella pneumoniae	-	1	2	9	16
0	1	No	Nosocomial	Klebsiella pneumoniae	-	1	2	9	16
0	2	Yes	Nosocomial	Klebsiella pneumoniae	-	1	2	9	16
3	1	No	Nosocomial	Klebsiella pneumoniae	-	2	2	6	7
0	1	No	Community-acquired	Klebsiella pneumoniae	-	1	2	9	16
0	1	Yes	Community-acquired	Klebsiella pneumoniae	-	1	1	7	13
3	1	Yes	Nosocomial	Klebsiella pneumoniae	-	1	2	9	16
0	1	Yes	Nosocomial	Klebsiella pneumoniae	-	1	2	9	16
2	1	Yes	Nosocomial	Klebsiella pneumoniae	-	1	2	9	16
2	3	Yes	Nosocomial	Klebsiella pneumoniae	-	1	2	9	16
0	1	Yes	Nosocomial	Klebsiella pneumoniae	-	1	1	4	4
0	2	Yes	Nosocomial	Klebsiella pneumoniae	-	1	0	5	7
2	2	No	Nosocomial	Klebsiella pneumoniae	-	1	1	7	11
1	2	Yes	Nosocomial	Klebsiella pneumoniae	-	1	1	7	11
2	1	Yes	Nosocomial	Klebsiella pneumoniae	-	0	1	6	10
2	4	Yes	Nosocomial	Klebsiella pneumoniae	-	0	1	6	10
2	2	Yes	Nosocomial	Klebsiella pneumoniae	-	0	1	6	10
0	1	Yes	Community-acquired	Klebsiella pneumoniae	-	0	1	6	10
2	2	Yes	Nosocomial	Klebsiella pneumoniae	-	0	1	6	10
2	1	Yes	Nosocomial	Klebsiella pneumoniae	-	1	2	2	1
0	2	Yes	Nosocomial	Klebsiella pneumoniae	-	1	2	5	5
0	2	Yes	Nosocomial	Klebsiella pneumoniae	-	1	2	5	5
2	1	No	Nosocomial	Klebsiella pneumoniae	-	1	2	2	1
0	1	Yes	Nosocomial	Klebsiella pneumoniae	-	1	2	2	2
2	3	Yes	Nosocomial	Klebsiella pneumoniae	-	1	2	5	5
0	2	Yes	Nosocomial	Klebsiella pneumoniae	-	0	0	1	0
0	2	Yes	Community-acquired	Klebsiella pneumoniae	-	1	1	6	10
2	2	Yes	Nosocomial	Klebsiella pneumoniae	-	1	1	3	3
1	1	No	Nosocomial	Klebsiella pneumoniae	-	1	1	4	3
0	1	Yes	Nosocomial	Klebsiella pneumoniae	-	1	1	6	10
2	1	No	Nosocomial	Klebsiella pneumoniae	-	1	1	6	10
2	2	Yes	Nosocomial	Klebsiella pneumoniae	-	1	1	6	10
1	1	Yes	Nosocomial	Klebsiella pneumoniae	-	1	1	6	10
0	2	Yes	Nosocomial	Klebsiella pneumoniae	-	1	0	0	0
3	1	Yes	Nosocomial	Klebsiella pneumoniae	-	1	0	0	0
3	2	Yes	Nosocomial	Klebsiella pneumoniae	-	1	0	7	11
3	2	Yes	Nosocomial	Klebsiella pneumoniae	-	0	1	6	9
0	2	Yes	Community-acquired	Klebsiella pneumoniae	-	1	1	9	13
0	1	Yes	Community-acquired	Klebsiella pneumoniae	-	0	1	6	7
0	1	Yes	Nosocomial	Klebsiella pneumoniae	-	1	2	2	2
3	1	Yes	Nosocomial	Klebsiella pneumoniae	-	1	2	2	2
3	1	Yes	Nosocomial	Klebsiella pneumoniae	-	1	2	2	2
2	2	Yes	Nosocomial	Klebsiella pneumoniae	-	1	2	2	2
1	1	Yes	Nosocomial	Klebsiella pneumoniae	-	1	2	3	3
3	2	Yes	Nosocomial	Klebsiella pneumoniae	-	1	2	2	2
0	3	Yes	Nosocomial	Klebsiella pneumoniae	-	1	2	2	2
0	2	Yes	Nosocomial	Klebsiella pneumoniae	-	1	2	2	2
2	1	Yes	Nosocomial	Klebsiella pneumoniae	-	1	2	2	2
0	1	No	Nosocomial	Klebsiella pneumoniae	-	1	2	2	2
3	3	Yes	Nosocomial	Klebsiella pneumoniae	-	1	2	2	2
0	3	Yes	Nosocomial	Klebsiella pneumoniae	-	1	2	2	2
0	3	Yes	Nosocomial	Klebsiella pneumoniae	-	1	2	2	2
0	1	No	Nosocomial	Klebsiella pneumoniae	-	1	2	2	2
1	2	Yes	Nosocomial	Klebsiella pneumoniae	-	1	2	2	2
2	1	Yes	Nosocomial	Klebsiella pneumoniae	-	1	2	2	2
0	2	Yes	Nosocomial	Klebsiella pneumoniae	-	1	2	2	2
2	3	Yes	Nosocomial	Klebsiella pneumoniae	-	1	2	1	1
1	1	No	Nosocomial	Klebsiella pneumoniae	total_size	1	2	6	8
3	3	Yes	Nosocomial	Klebsiella pneumoniae	-	1	2	2	2
1	2	Yes	Nosocomial	Klebsiella pneumoniae	-	1	2	2	2
1	2	Yes	Nosocomial	Klebsiella pneumoniae	-	1	2	2	2
0	1	Yes	Nosocomial	Klebsiella pneumoniae	-	1	2	2	2
0	1	Yes	Community-acquired	Klebsiella pneumoniae	-	1	0	1	1
1	2	Yes	Nosocomial	Klebsiella pneumoniae	-	1	2	2	2
3	2	Yes	Nosocomial	Klebsiella pneumoniae	-	1	2	2	2
1	1	Yes	Nosocomial	Klebsiella pneumoniae	-	1	2	2	2
3	1	Yes	Nosocomial	Klebsiella pneumoniae	-	1	2	2	2
1	3	No	Nosocomial	Klebsiella pneumoniae	-	1	2	2	2
1	3	Yes	Nosocomial	Klebsiella pneumoniae	-	1	2	2	2
2	2	Yes	Nosocomial	Klebsiella pneumoniae	-	1	2	2	2
0	2	Yes	Nosocomial	Klebsiella pneumoniae	-	1	2	2	2
4	2	Yes	Nosocomial	Klebsiella pneumoniae	-	1	0	1	1
0	1	No	Nosocomial	Klebsiella pneumoniae	-	0	0	5	6
2	2	Yes	Nosocomial	Klebsiella pneumoniae	-	0	1	9	14
1	1	No	Nosocomial	Klebsiella pneumoniae	-	0	1	9	13
0	2	Yes	Nosocomial	Klebsiella pneumoniae	-	0	1	8	12
0	1	No	Community-acquired	Klebsiella pneumoniae	-	0	1	9	13
0	1	Yes	Nosocomial	Klebsiella pneumoniae	-	1	2	8	12
0	2	Yes	Community-acquired	Klebsiella pneumoniae	-	1	1	6	8
3	1	Yes	Nosocomial	Klebsiella Ka3	-	0	0	0	0
3	1	Yes	Nosocomial	Klebsiella Ka3	-	0	0	0	0
0	2	Yes	Nosocomial	Klebsiella Ka3	-	0	0	0	0
0	1	No	Community-acquired	Klebsiella pneumoniae	-	1	0	0	0
0	1	No	Community-acquired	Klebsiella pneumoniae	-	1	1	2	2
0	2	No	Nosocomial	Klebsiella pneumoniae	-	0	0	0	0
0	2	No	Nosocomial	Klebsiella pneumoniae	-	0	0	0	0
1	1	No	Community-acquired	Klebsiella pneumoniae	total_size	0	0	2	2
3	1	Yes	Nosocomial	Klebsiella pneumoniae	-	1	1	8	17
1	2	Yes	Nosocomial	Klebsiella pneumoniae	-	3	0	1	1
0	1	Yes	Community-acquired	Klebsiella pneumoniae	-	0	0	0	0
0	1	No	Community-acquired	Klebsiella pneumoniae	-	0	0	0	0
0	2	Yes	Nosocomial	Klebsiella pneumoniae	-	0	0	0	0
0	1	No	Nosocomial	Klebsiella pneumoniae	-	1	0	0	0
0	1	Yes	Community-acquired	Klebsiella pneumoniae	-	0	0	0	0
3	1	Yes	Nosocomial	Klebsiella pneumoniae	-	0	0	0	0
0	1	Yes	Nosocomial	Klebsiella pneumoniae	-	0	0	0	0
4	3	Yes	Nosocomial	Klebsiella pneumoniae	-	0	0	0	0
1	3	Yes	Nosocomial	Klebsiella variicola subsp. variicola	-	0	0	0	0
3	1	Yes	Community-acquired	Klebsiella variicola subsp. variicola	-	0	0	0	0
3	2	Yes	Nosocomial	Klebsiella variicola subsp. variicola	-	0	2	1	1
1	2	No	Nosocomial	Klebsiella variicola subsp. variicola	total_size	0	2	5	5
				Klebsiella pneumoniae	-			1	0

KLEBORATE	KLEBORATE	KLEBORATE	KLEBORATE	KLEBORATE	KLEBORATE	KLEBORATE	KLEBORATE	KLEBORATE	KLEBORATE	KLEBORATE	KLEBORATE
**Yersiniabactin**	**YbST**	**Colibactin**	**CbST**	**Aerobactin**	**AbST**	**Salmochelin**	**SmST**	**wzi**	**K locus**	**K type**	**K locus missing genes**
-	0	-	0	-	0	-	0	wzi93	KL112	unknown (KL112)	
-	0	-	0	-	0	-	0	wzi93	KL112	unknown (KL112)	
-	0	-	0	-	0	-	0	wzi93	KL112	unknown (KL112)	
ybt 10; ICEKp4	445-3LV	-	0	-	0	-	0	-	KL23	K23	
ybt 10; ICEKp4	445-3LV	-	0	-	0	-	0	-	KL23	K23	
ybt 10; ICEKp4	445-3LV	-	0	-	0	-	0	-	KL23	K23	
ybt 10; ICEKp4	445-3LV	-	0	-	0	-	0	-	KL23	K23	KL23_04_wza
-	0	-	0	-	0	-	0	-	KL23	K23	
-	0	-	0	-	0	-	0	-	KL23	K23	
-	0	-	0	-	0	-	0	-	unknown (KL52)	unknown (K52)	3_rmlB,KL52_19_rmlA,KL52_20_rmlD,KL52_
-	0	-	0	-	0	-	0	-	unknown (KL52)	unknown (K52)	3_rmlB,KL52_19_rmlA,KL52_20_rmlD,KL52_
ybt 10; ICEKp4	107	-	0	-	0	-	0	-	KL57	K57	KL57_13_vanT
ybt 10; ICEKp4	107	-	0	-	0	-	0	-	KL57	K57	KL57_13_vanT
ybt 10; ICEKp4	107	-	0	-	0	-	0	-	KL57	K57	KL57_13_vanT
ybt 10; ICEKp4	107	-	0	-	0	-	0	-	KL57	K57	KL57_13_vanT
ybt 10; ICEKp4	107	-	0	-	0	-	0	-	KL57	K57	KL57_13_vanT
ybt 10; ICEKp4	107	-	0	-	0	-	0	-	KL57	K57	KL57_13_vanT
ybt 17; ICEKp10	289	clb 3	17-1LV	-	0	-	0	wzi40	KL3	K3	
ybt 10; ICEKp4 (truncated)	107-1LV	-	0	-	0	-	0	-	KL57	K57	KL57_13_vanT
ybt 10; ICEKp4	107	-	0	-	0	-	0	-	KL57	K57	KL57_13_vanT
ybt 10; ICEKp4	107	-	0	-	0	-	0	-	KL57	K57	KL57_13_vanT
ybt 10; ICEKp4	107	-	0	-	0	-	0	-	KL57	K57	KL57_13_vanT
ybt 10; ICEKp4	107	-	0	-	0	-	0	-	KL57	K57	KL57_13_vanT
ybt 10; ICEKp4	107	-	0	-	0	-	0	-	KL57	K57	KL57_13_vanT
ybt 5; ICEKp6	10-1LV	-	0	-	0	-	0	wzi37	KL22	K22	
ybt 5; ICEKp6	10-1LV	-	0	-	0	-	0	wzi37	KL22	K22	
ybt 27; ICEKp22	312	-	0	-	0	-	0	wzi143	KL151	unknown (KL151)	
ybt 27; ICEKp22	312	-	0	-	0	-	0	wzi143	KL151	unknown (KL151)	
-	0	-	0	-	0	-	0	wzi160	KL39	K39	
-	0	-	0	-	0	-	0	wzi160	KL39	K39	KL39_16_wcaJ
-	0	-	0	-	0	-	0	wzi160	KL39	K39	
-	0	-	0	-	0	-	0	wzi160	KL39	K39	
-	0	-	0	-	0	-	0	wzi160	KL39	K39	
ybt 16; ICEKp12	277-1LV	-	0	-	0	-	0	wzi64	KL64	K64	
ybt 16; ICEKp12	277-3LV	-	0	-	0	-	0	wzi64	KL64	K64	
ybt 16; ICEKp12	277-3LV	-	0	-	0	-	0	wzi64	KL64	K64	
ybt 16; ICEKp12	277-2LV	-	0	-	0	-	0	wzi64	KL64	K64	
ybt 16; ICEKp12	277-3LV	-	0	-	0	-	0	wzi64	KL64	K64	
ybt 16; ICEKp12	277-3LV	-	0	-	0	-	0	wzi64	KL64	K64	
-	0	-	0	-	0	-	0	wzi64	KL64	K64	
ybt 14; ICEKp5	151	-	0	-	0	-	0	wzi19	KL19	K19	
ybt 14; ICEKp5	151	-	0	-	0	-	0	wzi19	KL19	K19	
ybt 14; ICEKp5	151	-	0	-	0	-	0	wzi19	KL19	K19	
ybt 14; ICEKp5	151	-	0	-	0	-	0	wzi19	KL19	K19	
ybt 14; ICEKp5	151	-	0	-	0	-	0	wzi19	KL19	K19	
ybt 14; ICEKp5	151	-	0	-	0	-	0	wzi19	KL19	K19	
ybt 14; ICEKp5	151	-	0	-	0	-	0	wzi19	KL19	K19	
ybt 15; ICEKp11	378	-	0	-	0	-	0	wzi96	KL38	K38	
ybt 15; ICEKp11	378	-	0	-	0	-	0	wzi96	KL38	K38	
ybt 10; ICEKp4	78	-	0	-	0	-	0	wzi101	KL24	K24	KL24_13_wcuG
-	0	-	0	-	0	-	0	wzi101	KL24	K24	
ybt 10; ICEKp4	78	-	0	-	0	-	0	wzi101	KL24	K24	
-	0	-	0	-	0	-	0	wzi101	KL24	K24	
ybt 10; ICEKp4	78-1LV	-	0	-	0	-	0	wzi149	KL62	K62	
ybt 10; ICEKp4	78-1LV	-	0	-	0	-	0	wzi149	KL62	K62	
ybt 10; ICEKp4	78-1LV	-	0	-	0	-	0	wzi149	KL62	K62	
ybt 10; ICEKp4	78-1LV	-	0	-	0	-	0	wzi149	KL62	K62	
ybt 10; ICEKp4	78-1LV	-	0	-	0	-	0	wzi149	KL62	K62	
ybt 10; ICEKp4	78-1LV	-	0	-	0	-	0	wzi149	KL62	K62	
ybt 10; ICEKp4	78-1LV	-	0	-	0	-	0	wzi149	KL62	K62	
ybt 10; ICEKp4	78-1LV	-	0	-	0	-	0	wzi149	KL62	K62	
ybt 10; ICEKp4	78-1LV	-	0	-	0	-	0	wzi149	KL62	K62	
ybt 10; ICEKp4	78-1LV	-	0	-	0	-	0	wzi149	KL62	K62	
ybt 10; ICEKp4	78-1LV	-	0	-	0	-	0	wzi149	KL62	K62	
ybt 10; ICEKp4	78-1LV	-	0	-	0	-	0	wzi149	KL62	K62	
ybt 10; ICEKp4	78-1LV	-	0	-	0	-	0	wzi149	KL62	K62	
ybt 10; ICEKp4	78-1LV	-	0	-	0	-	0	wzi149	KL62	K62	
ybt 10; ICEKp4	78-1LV	-	0	-	0	-	0	wzi149	KL62	K62	
ybt 10; ICEKp4	78-1LV	-	0	-	0	-	0	wzi149	KL62	K62	
ybt 10; ICEKp4	78-1LV	-	0	-	0	-	0	wzi149	KL62	K62	
ybt 10; ICEKp4	78-1LV	-	0	-	0	-	0	wzi149	KL62	K62	
ybt 10; ICEKp4	78-1LV	-	0	-	0	-	0	wzi149	KL62	K62	
ybt 10; ICEKp4	78-1LV	-	0	-	0	-	0	wzi149	KL62	K62	
ybt 10; ICEKp4	78-1LV	-	0	-	0	-	0	wzi149	KL62	K62	
ybt 10; ICEKp4	78-1LV	-	0	-	0	-	0	wzi149	KL62	K62	
ybt 10; ICEKp4	78-1LV	-	0	-	0	-	0	wzi149	KL62	K62	
ybt 10; ICEKp4	78-1LV	-	0	-	0	-	0	wzi149	KL62	K62	
ybt 10; ICEKp4	78-1LV	-	0	-	0	-	0	wzi149	KL62	K62	
ybt 10; ICEKp4	78-1LV	-	0	-	0	-	0	wzi149	KL62	K62	
ybt 10; ICEKp4	78-1LV	-	0	-	0	-	0	wzi149	KL62	K62	
ybt 10; ICEKp4	78-1LV	-	0	-	0	-	0	wzi149	KL62	K62	
ybt 10; ICEKp4	78-1LV	-	0	-	0	-	0	wzi149	KL62	K62	
ybt 10; ICEKp4	78-1LV	-	0	-	0	-	0	wzi149	KL62	K62	
ybt 10; ICEKp4	78-1LV	-	0	-	0	-	0	wzi149	KL62	K62	
ybt 10; ICEKp4	78-1LV	-	0	-	0	-	0	wzi149	KL62	K62	
ybt 10; ICEKp4	78-1LV	-	0	-	0	-	0	wzi149	KL62	K62	
ybt 10; ICEKp4	78-1LV	-	0	-	0	-	0	wzi149	KL62	K62	
ybt 10; ICEKp4	78-1LV	-	0	-	0	-	0	wzi149	KL62	K62	
-	0	-	0	-	0	-	0	wzi173	KL102	unknown (KL102)	
-	0	-	0	-	0	-	0	wzi173	KL102	unknown (KL102)	
-	0	-	0	-	0	-	0	wzi173	KL102	unknown (KL102)	
-	0	-	0	-	0	-	0	wzi173	KL102	unknown (KL102)	
-	0	-	0	-	0	-	0	wzi173	KL102	unknown (KL102)	
ybt 0; ICEKp5	522-2LV	-	0	-	0	-	0	-	KL62	K62	
ybt 0; ICEKp5	522-2LV	-	0	-	0	-	0	wzi94	KL62	K62	
-	0	-	0	-	0	iro unknown	0	-	unknown (KL167)	unknown (KL167)	9_wzx,KL167_10_wckK,KL167_11_manC,KL
-	0	-	0	-	0	iro unknown	0	-	unknown (KL167)	unknown (KL167)	9_wzx,KL167_10_wckK,KL167_11_manC,KL
-	0	-	0	-	0	iro unknown	0	-	unknown (KL167)	unknown (KL167)	9_wzx,KL167_10_wckK,KL167_11_manC,KL
ybt 9; ICEKp3	183-2LV	-	0	-	0	-	0	wzi117	KL54	K54	
ybt 4; plasmid	463-2LV	-	0	-	0	-	0	wzi5	KL5	K5	KL5_17_manC,KL5_18_manB
-	0	-	0	-	0	-	0	wzi112	KL13	K13	KL13_17_manC,KL13_18_manB
-	0	-	0	-	0	-	0	wzi342	KL109	unknown (KL109)	
-	0	-	0	-	0	-	0	wzi63	KL63	K63	
ybt 10; ICEKp4	445-4LV	-	0	-	0	-	0	wzi24	KL24	K24	
-	0	-	0	iuc 5	62-1LV	iro 5	34-1LV	wzi123	KL136	unknown (KL136)	
-	0	-	0	-	0	-	0	wzi177	KL125	unknown (KL125)	
-	0	-	0	-	0	-	0	wzi38	KL38	K38	
-	0	-	0	-	0	-	0	-	KL48	K48	
ybt 4; plasmid	31	-	0	-	0	-	0	wzi149	KL62	K62	
-	0	-	0	-	0	-	0	wzi360	KL122	unknown (KL122)	KL122_15_wcaJ
-	0	-	0	-	0	-	0	wzi227	KL60	K60	
-	0	-	0	-	0	-	0	wzi374	KL140	unknown (KL140)	
-	0	-	0	-	0	-	0	wzi150	KL46	K46	KL46_19_manB
-	0	-	0	-	0	-	0	wzi542	unknown (KL55)	unknown (K55)	_14,KL55_16_rmlB,KL55_17_rmlA,KL55_19_
-	0	-	0	-	0	-	0	wzi53	KL53	K53	KL53_18_manB
-	0	-	0	-	0	-	0	wzi532	KL9	K9	
-	0	-	0	-	0	-	0	wzi532	KL9	K9	
-	0	-	0	-	0	-	0	wzi50	KL52	K52	

KLEBORATE	KLEBORATE	KLEBORATE	KLEBORATE	KLEBORATE	KLEBORATE	KLEBORATE	KLEBORATE	KLEBORATE
**O_locus**	**O_type**	**O locus missing genes**	**Agly acquired**	**Flq acquired**	**MLS acquired**	**Phe acquired**	**Rif acquired**	**Sul acquired**
O1/O2v1	O1		aac(6')-Ib-cr.v2	qnrB1.v2^	mphA	catA1^	-	sul1
O1/O2v1	O1		aac(6')-Ib-cr.v2	qnrB1.v2^	mphA	catA1^	-	sul1
O1/O2v1	O1		aac(6')-Ib-cr.v2;aadA2^	qnrB1.v2^	mphA	catA1^	-	sul1
O1/O2v2	O1		aac(3)-IIa.v1;aac(6')-Ib-cr.v2;aadA16*	qnrB6^	-	catA1^	arr-3	sul1;sul1
O1/O2v2	O1		aac(3)-IIa.v1;aac(6')-Ib-cr.v2;aadA16*	-	-	catA1^	arr-3	sul1
O1/O2v2	O1		aac(3)-IIa.v1;aac(6')-Ib-cr.v2;aadA16*	qnrB6^	-	catA1^	arr-3	sul1;sul1
O1/O2v2	O1		aac(3)-IIa.v1;aac(6')-Ib-cr.v2;aadA16*	qnrB6^	-	catA1^	arr-3	sul1;sul1
O1/O2v2	O1		aac(3)-IIa.v1;aac(6')-Ib-cr.v2;aadA16*	qnrB6^	-	catA1^	arr-3	sul1;sul1
O1/O2v2	O1		aac(3)-IIa.v1;aac(6')-Ib-cr.v2;aadA16*	-	-	catA1^	arr-3	sul1
OL103	unknown (OL103)		-	-	-	-	-	sul2*
OL103	unknown (OL103)		-	-	-	-	-	sul2*
O1/O2v2	O1		aac(3)-IIa.v1^;aac(6')-Ib-cr.v2;aadA2^;strA.v1*;strB.v1*	qnrS1	mphA	-	-	sul1;sul2
O1/O2v2	O1		aac(3)-IIa.v1^;aac(6')-Ib-cr.v2;aadA2^;strA.v1*;strB.v1*	qnrS1	mphA	-	-	sul1;sul2
O1/O2v2	O1		aac(3)-IIa.v1^;aac(6')-Ib-cr.v2;aadA2^;strA.v1*;strB.v1*	qnrS1	mphA	-	-	sul1;sul2
O1/O2v2	O1		aac(3)-IIa.v1^;aac(6')-Ib-cr.v2;aadA2^;strA.v1*;strB.v1*	qnrS1	mphA	-	-	sul1;sul2
O1/O2v2	O1		aac(3)-IIa.v1^;aac(6')-Ib-cr.v2;aadA2^;strA.v1*;strB.v1*	qnrS1	mphA	-	-	sul1;sul2
O1/O2v2	O1		aac(3)-IIa.v1^;aac(6')-Ib-cr.v2;aadA2^;strA.v1*;strB.v1*	qnrS1	mphA	-	-	sul1;sul2
O1/O2v2	O1		aadA*;strA.v1^;strB.v1	-	-	-	-	sul2
O1/O2v2	O2afg		aac(3)-IIa.v1^;aac(6')-Ib-cr.v2;aadA2^;strA.v1*;strB.v1*	qnrS1	mphA	-	-	sul1;sul2
O1/O2v2	O1		aac(3)-IIa.v1^;aac(6')-Ib-cr.v2;aadA2^;strA.v1*;strB.v1*	qnrS1	mphA	-	-	sul1;sul2
O1/O2v2	O1		aac(3)-IIa.v1^;aac(6')-Ib-cr.v2;aadA2^;strA.v1*;strB.v1*	qnrS1	mphA	-	-	sul1;sul2
O1/O2v2	O1		aac(3)-IIa.v1^;aac(6')-Ib-cr.v2;aadA2^;strA.v1*;strB.v1*	qnrS1	mphA	-	-	sul1;sul2
O1/O2v2	O1		aac(3)-IIa.v1^;aac(6')-Ib-cr.v2;aadA2^;strA.v1*;strB.v1*	qnrS1	mphA	-	-	sul1;sul2
O1/O2v2	O1		aac(3)-IIa.v1^;aac(6')-Ib-cr.v2;aadA2^;strA.v1*;strB.v1*	qnrS1	mphA	-	-	sul1;sul2
O1/O2v1	O1		aac(3)-IIa.v1	-	-	catA1^	-	-
O1/O2v1	O1		-	qnrS1	-	-	-	sul1;sul2
O4	O4		aac(3)-IIa.v1^;aac(6')-Ib-cr.v2;strA.v1^;strB.v1	qnrB1.v2^	-	-	-	sul2
O4	O4		aac(3)-IIa.v1^;aac(6')-Ib-cr.v2;strA.v1^;strB.v1	qnrB1.v2^	-	-	-	sul2
O1/O2v1	O1		aac(3)-IIa.v1^;aac(6')-Ib-cr.v2;strA.v1^;strB.v1	qnrB1.v2^	-	-	-	sul2
O1/O2v1	O1		aac(3)-IIa.v1^;aac(6')-Ib-cr.v2*;strA.v1^;strB.v1	qnrB1.v2^	-	-	-	sul2
O1/O2v1	O1		aac(3)-IIa.v1^;aac(6')-Ib-cr.v2;strA.v1^;strB.v1	qnrB1.v2^	-	-	-	sul2
O1/O2v1	O1		aac(3)-IIa.v1^;aac(6')-Ib-cr.v2;strA.v1^;strB.v1	qnrB1.v2^	-	-	-	sul2
O1/O2v1	O1		aac(3)-IIa.v1^;aac(6')-Ib-cr.v2;strA.v1^;strB.v1	qnrB1.v2^	-	-	-	sul2
O1/O2v1	O2a		-	-	-	-	-	-
O1/O2v1	O2a		aph3-Ia.v1^	qnrS1	-	-	-	sul2
O1/O2v1	O2a		aph3-Ia.v1^	qnrS1	-	-	-	sul2
O1/O2v1	O2a		-	-	-	-	-	-
O1/O2v1	O2a		-	qnrS1	-	-	-	-
O1/O2v1	O2a		aph3-Ia.v1^	qnrS1	-	-	-	sul2
O1/O2v1	O2a		-	-	-	-	-	-
O1/O2v2	O1		aac(3)-IIa.v1^;aac(6')-Ib-cr.v2;strA.v1^;strB.v1	qnrB1.v2^	-	-	-	sul2
O1/O2v2	O1		-	qnrB1.v2^	-	-	-	-
O1/O2v2	O1		-	qnrB1.v2^	-	-	-	-
O1/O2v2	O1		aac(3)-IIa.v1^;aac(6')-Ib-cr.v2;strA.v1^;strB.v1	qnrB1.v2^	-	-	-	sul2
O1/O2v2	O1		aac(3)-IIa.v1^;aac(6')-Ib-cr.v2;strA.v1^;strB.v1	qnrB1.v2^	-	-	-	sul2
O1/O2v2	O1		aac(3)-IIa.v1^;aac(6')-Ib-cr.v2;strA.v1^;strB.v1	qnrB1.v2^	-	-	-	sul2
O1/O2v2	O1		aac(3)-IIa.v1^;aac(6')-Ib-cr.v2;strA.v1^;strB.v1	qnrB1.v2^	-	-	-	sul2
O3b	O3b		-	-	-	-	-	-
O3b	O3b		-	-	-	-	-	-
O1/O2v1	O2a		aac(6')-Ib-cr.v2;aadA16*?;strA.v1*;strB.v1*	qnrB6^	-	catII.2*	arr-3	sul1;sul2
O1/O2v1	O2a		aac(6')-Ib-cr.v2;strA.v1^;strB.v1*	qnrB1.v2^	-	-	-	sul2
O1/O2v1	O2a		aac(6')-Ib-cr.v2;aadA16*?;strA.v1*;strB.v1*	qnrB6^	-	catII.2*	arr-3	sul1;sul2
O1/O2v1	O2a		strA.v1^;strB.v1*	qnrB1.v2^	-	-	-	sul2
O1/O2v1	O2a		-	-	-	-	-	-
O1/O2v1	O2a		-	-	-	-	-	-
O1/O2v1	O2a		-	-	-	-	-	-
O1/O2v1	O2a		-	-	-	-	-	-
O1/O2v1	O2a		-	qnrS1	-	-	-	-
O1/O2v1	O2a		-	-	-	-	-	-
O1/O2v1	O2a		-	-	-	-	-	-
O1/O2v1	O2a		-	-	-	-	-	-
O1/O2v1	O2a		-	-	-	-	-	-
O1/O2v1	O2a		-	-	-	-	-	-
O1/O2v1	O2a	O1/O2v1_06_wbbO	-	-	-	-	-	-
O1/O2v1	O2a		-	-	-	-	-	-
O1/O2v1	O2a		-	-	-	-	-	-
O1/O2v1	O2a		-	-	-	-	-	-
O1/O2v1	O2a		-	-	-	-	-	-
O1/O2v1	O2a		-	-	-	-	-	-
O1/O2v1	O2a		-	-	-	-	-	-
O1/O2v1	O2a		-	-	-	-	-	-
O1/O2v1	O2a		aph(3')-IIb*	crpP*;crpP*	-	catB7	-	-
O1/O2v1	O2a		-	-	-	-	-	-
O1/O2v1	O2a		-	-	-	-	-	-
O1/O2v1	O2a		-	-	-	-	-	-
O1/O2v1	O2a		-	-	-	-	-	-
O1/O2v1	O2a		-	-	-	-	-	-
O1/O2v1	O2a		-	-	-	-	-	-
O1/O2v1	O2a		-	-	-	-	-	-
O1/O2v1	O2a		-	-	-	-	-	-
O1/O2v1	O2a		-	-	-	-	-	-
O1/O2v1	O2a		-	-	-	-	-	-
O1/O2v1	O2a		-	-	-	-	-	-
O1/O2v1	O2a		-	-	-	-	-	-
O1/O2v1	O2a		-	-	-	-	-	-
O1/O2v1	O2a	O1/O2v1_06_wbbO	-	-	-	-	-	-
O1/O2v2	O2afg		aac(6')-Ib-cr.v2;aadA16*?	qnrB6^	-	-	arr-3	sul1
O1/O2v2	O2afg		aac(3)-IIa.v1;aac(6')-Ib-cr.v2;aadA16*?;strA.v1*;strB.v1*	qnrB6^	-	catII.2*	arr-3	sul1;sul2
O1/O2v2	O2afg		aac(6')-Ib-cr.v2;aadA16*?;strA.v1*;strB.v1*	qnrB6^	-	catII.2*	arr-3	sul1;sul2
O1/O2v2	O2afg		aac(6')-Ib-cr.v2;aadA16*?;strA.v1*;strB.v1*	qnrB6^	-	catII.2*	arr-3	sul1;sul2
unknown (O1/O2v2)	unknown (O2afg)	bbM,O1/O2v2_04_glf,O1/O2	aac(6')-Ib-cr.v2;aadA16*?;strA.v1*;strB.v1*	qnrB6^	-	catII.2*	arr-3	sul1;sul2
O1/O2v1	O1		aac(3)-IIa.v1^;aac(6')-Ib-cr.v2;strA.v1^;strB.v1	qnrB1.v2^	-	-	-	sul2
O1/O2v1	O1		aac(3)-IIa.v1^;strA.v1^;strB.v1	qnrB1.v2^	-	-	-	sul2
unknown (O1/O2v1)	unknown (O2a)	2v1_04_glf,O1/O2v1_05_wb	-	-	-	-	-	-
unknown (O1/O2v1)	unknown (O2a)	2v1_04_glf,O1/O2v1_05_wb	-	-	-	-	-	-
unknown (O1/O2v1)	unknown (O2a)	2v1_04_glf,O1/O2v1_05_wb	-	-	-	-	-	-
O1/O2v1	O1		-	-	-	-	-	-
O3b	O3b	3b_01_manC,O3b_02_man	-	qnrS1	-	-	-	-
O3b	O3b	3b_01_manC,O3b_02_man	-	-	-	-	-	-
O1/O2v2	O2afg		-	-	-	-	-	-
O1/O2v2	O1		-	-	-	catA4*	-	-
O1/O2v1	O1		aac(3)-IIa.v1^;aac(3)-IId^;aac(6')-Ib-cr.v2;aadA1.v1^;aadA16*;strA.v1^;strB.v1	qnrB6^	-	catA1^	arr-3	sul1;sul2
O1/O2v2	O2afg		-	-	-	-	-	-
O5	O5		-	-	-	-	-	-
O1/O2v2	O1		-	-	-	-	-	-
O3/O3a	O3/O3a		-	-	-	-	-	-
O1/O2v1	O1		-	-	-	-	-	-
O1/O2v2	O2afg		-	-	-	-	-	-
O3b	O3b		-	-	-	-	-	-
O1/O2v2	O1		-	-	-	-	-	-
O3b	O3b	O3b_02_manB	-	-	-	-	-	-
OL103	unknown (OL103)		-	-	-	-	-	-
O3/O3a	O3/O3a	O3/O3a_02_manB	-	-	-	-	-	-
O3/O3a	O3/O3a		-	-	-	-	-	-
O3/O3a	O3/O3a		aph(3')-IIb*	-	-	catB7*	-	-
OL101	unknown (OL101)		-	-	-	-	-	-

KLEBORATE	KLEBORATE	KLEBORATE	KLEBORATE	KLEBORATE	KLEBORATE	KLEBORATE	KLEBORATE	KLEBORATE	KLEBORATE
**Tet acquired**	**Tmt acquired**	**Bla acquired**	**Bla ESBL acquired**	**Bla Carb acquired**	**Bla chr**	**SHV mutations**	**Omp mutations**	**Col mutations**	**Flq mutations**
-	dfrA14.v2*	OXA-1	CTX-M-15	-	SHV-28.v1^	-	-	-	GyrA-83F;GyrA-87A;ParC-80I
-	dfrA14.v2*	OXA-1	CTX-M-15	-	SHV-28.v1^	-	-	-	GyrA-83F;GyrA-87A;ParC-80I
-	dfrA12;dfrA14.v2*	OXA-1	CTX-M-15	-	SHV-28.v1^	-	-	-	GyrA-83F;GyrA-87A;ParC-80I
-	dfrA27	OXA-1;TEM-1D.v1^	CTX-M-15	-	SHV-28.v1^	-	-	-	GyrA-83F;GyrA-87A;ParC-80I
-	dfrA27	OXA-1;TEM-1D.v1^	CTX-M-15	-	SHV-28.v1^	-	-	-	GyrA-83F;GyrA-87A;ParC-80I
-	dfrA27	OXA-1;TEM-1D.v1^	CTX-M-15	-	SHV-28.v1^	-	-	-	GyrA-83F;GyrA-87A;ParC-80I
-	dfrA27	OXA-1;TEM-1D.v1^	CTX-M-15	-	SHV-28.v1^	-	-	-	GyrA-83F;GyrA-87A;ParC-80I
-	dfrA27	OXA-1;TEM-1D.v1^	CTX-M-15	-	SHV-28.v1^	-	-	-	GyrA-83F;GyrA-87A;ParC-80I
-	dfrA27	OXA-1;TEM-1D.v1^	CTX-M-15	-	SHV-28.v1^	-	-	-	GyrA-83F;GyrA-87A;ParC-80I
-	-	-	-	-	SHV-1^	-	OmpK35-32%	-	-
-	-	-	-	-	SHV-1^	-	-	-	-
tet(A).v1	dfrA12;dfrA14.v2*	OXA-1;TEM-1D.v1^	CTX-M-15	KPC-3	SHV-1^	-	-	-	-
tet(A).v1	dfrA12;dfrA14.v2*	OXA-1;TEM-1D.v1^	CTX-M-15	KPC-3	SHV-1^	-	-	-	-
tet(A).v1	dfrA12;dfrA14.v2*	OXA-1;TEM-1D.v1^	CTX-M-15	KPC-3	SHV-1^	-	-	-	-
tet(A).v1	dfrA12;dfrA14.v2*	OXA-1;TEM-1D.v1^	CTX-M-15	KPC-3	SHV-1^	-	-	-	-
tet(A).v1	dfrA12;dfrA14.v2*	OXA-1;TEM-1D.v1^	CTX-M-15	KPC-3	SHV-1^	-	-	-	-
tet(A).v1	dfrA12;dfrA14.v2*	OXA-1;TEM-1D.v1^	CTX-M-15	KPC-3	SHV-1^	-	-	-	-
-	dfrA14.v2*	OXA-9.v1	-	KPC-3	SHV-1^	-	OmpK35-70%	-	GyrA-87N
tet(A).v1	dfrA12;dfrA14.v2*	OXA-1;TEM-1D.v1^	CTX-M-15	KPC-3	SHV-1^	-	OmpK36-0%	-	-
-	dfrA12	OXA-1;TEM-1D.v1^	CTX-M-15	-	SHV-1^	-	-	-	-
tet(A).v1	dfrA12;dfrA14.v2*	OXA-1;TEM-1D.v1^	CTX-M-15	KPC-3	SHV-1^	-	-	-	-
tet(A).v1	dfrA12;dfrA14.v2*	OXA-1;TEM-1D.v1^	CTX-M-15	KPC-3	SHV-1^	-	-	-	-
tet(A).v1	dfrA12;dfrA14.v2*	OXA-1;TEM-1D.v1^	CTX-M-15	KPC-3	SHV-1^	-	-	-	-
tet(A).v1	dfrA12;dfrA14.v2*	OXA-1;TEM-1D.v1^	CTX-M-15	KPC-3	SHV-1^	-	-	-	-
-	-	TEM-1D.v1^	CTX-M-15	-	SHV-33	-	-	-	-
tet(A).v1*;tet(D)	dfrA25	LAP-2	-	-	-	-	-	-	-
tet(A).v1	dfrA14.v2*	OXA-1;TEM-1D.v1^	CTX-M-15	-	SHV-76	35Q	-	-	-
tet(A).v1	dfrA14.v2*	OXA-1;TEM-1D.v1^	CTX-M-15	-	SHV-76	35Q	-	-	-
-	dfrA14.v2*	OXA-1;TEM-1D.v1^	CTX-M-15	-	SHV-187	-	-	-	-
-	dfrA14.v2*	OXA-1;TEM-1D.v1^	CTX-M-15	-	SHV-187	-	-	-	-
-	dfrA14.v2*	OXA-1;TEM-1D.v1^	CTX-M-15	-	SHV-187	-	-	-	-
-	dfrA14.v2*	OXA-1;TEM-1D.v1^	CTX-M-15	-	SHV-187	-	-	-	-
-	dfrA14.v2*	OXA-1;TEM-1D.v1^	CTX-M-15	-	SHV-187	-	-	-	-
-	-	-	-	KPC-3	SHV-11.v1^	35Q	-	-	GyrA-83I;ParC-80I
tet(D)	-	-	-	KPC-3	SHV-11.v1^	35Q	-	-	GyrA-83I;ParC-80I
tet(D)	-	-	-	KPC-3	SHV-11.v1^	35Q	-	-	GyrA-83I;ParC-80I
-	-	-	-	KPC-3	SHV-11.v1^	35Q	-	-	GyrA-83I;ParC-80I
-	-	-	-	KPC-3	SHV-11.v1^	35Q	-	-	GyrA-83I;ParC-80I
tet(D)	-	-	-	KPC-3	SHV-11.v1^	35Q	-	-	GyrA-83I;ParC-80I
-	-	-	-	-	SHV-11.v1^	35Q	-	-	GyrA-83I;ParC-80I
-	dfrA14.v2*	OXA-1;TEM-1D.v1^	CTX-M-15	-	-	-	-	-	-
-	dfrA14.v2*	-	CTX-M-15	-	-	-	-	-	-
-	dfrA14.v2*	-	CTX-M-15	-	-	-	-	PmrB-0%	-
-	dfrA14.v2*	OXA-1;TEM-1D.v1^	CTX-M-15	-	-	-	-	-	-
-	dfrA14.v2*	OXA-1;TEM-1D.v1^	CTX-M-15	-	-	-	-	-	-
-	dfrA14.v2*	OXA-1;TEM-1D.v1^	CTX-M-15	-	-	-	-	-	-
-	dfrA14.v2*	OXA-1;TEM-1D.v1^	CTX-M-15	-	-	-	-	-	-
-	-	-	-	-	SHV-11.v1^	35Q	-	-	-
-	-	-	-	-	SHV-11.v1^	35Q	-	-	-
-	dfrA27	TEM-1D.v1^	-	-	SHV-1	-	-	-	-
-	dfrA14.v2*	OXA-1;TEM-1D.v1^	CTX-M-15	-	-	-	-	-	-
tet(D)	dfrA27	TEM-1D.v1^	CTX-M-15	-	SHV-1	-	-	-	-
-	dfrA14.v2*	TEM-1D.v1^	CTX-M-15	-	-	-	-	-	-
tet(D)	-	-	-	KPC-3	SHV-1	-	-	-	-
tet(D)	-	-	-	KPC-3	-	-	-	-	-
tet(D)	-	-	-	KPC-3	SHV-1^	-	-	-	-
tet(D)	-	-	-	KPC-3	-	-	-	-	-
tet(D)	-	-	-	KPC-3	-	-	-	-	-
tet(D)	-	-	-	KPC-3	-	-	-	-	-
tet(D)	-	-	-	KPC-3	-	-	-	-	-
tet(D)	-	-	-	KPC-3	-	-	-	-	-
tet(D)	-	-	-	KPC-3	-	-	-	-	-
tet(D)	-	-	-	KPC-3	-	-	-	-	-
tet(D)	-	-	-	KPC-3	-	-	-	-	-
tet(D)	-	-	-	KPC-3	-	-	-	-	-
tet(D)	-	-	-	KPC-3	-	-	-	-	-
tet(D)	-	-	-	KPC-3	-	-	-	-	-
tet(D)	-	-	-	KPC-3	SHV-1	-	-	-	-
tet(D)	-	-	-	KPC-3	-	-	-	-	-
tet(D)	-	-	-	KPC-3	-	-	-	-	-
-	-	-	-	KPC-3	SHV-1	-	OmpK35-64%	-	-
tet(D)	-	OXA-486*;PDC-1^	-	KPC-3	SHV-1^	-	-	-	-
tet(D)	-	-	-	KPC-3	SHV-1^	-	-	-	-
tet(D)	-	-	-	KPC-3	-	-	-	-	-
tet(D)	-	-	-	KPC-3	-	-	-	-	-
tet(D)	-	-	-	KPC-3	-	-	-	-	-
tet(D)	-	-	-	-	SHV-1^	-	-	-	-
tet(D)	-	-	-	KPC-3	-	-	-	-	-
tet(D)	-	-	-	KPC-3	-	-	-	-	-
tet(D)	-	-	-	KPC-3	-	-	-	-	-
tet(D)	-	-	-	KPC-3	-	-	-	-	-
tet(D)	-	-	-	KPC-3	SHV-1	-	-	-	-
tet(D)	-	-	-	KPC-3	-	-	-	-	-
tet(D)	-	-	-	KPC-3	-	-	-	-	-
tet(D)	-	-	-	KPC-3	-	-	-	-	-
tet(D)	-	-	-	-	-	-	-	-	-
-	dfrA27	-	-	-	SHV-28.v1^	-	OmpK35-32%	-	GyrA-83I;ParC-80I
tet(D)	dfrA27	TEM-1D.v1^	CTX-M-15	-	SHV-28.v1^	-	OmpK35-32%	-	GyrA-83I;ParC-80I
tet(D)	dfrA27	TEM-1D.v1^	CTX-M-15	-	SHV-28.v1^	-	OmpK35-32%	-	GyrA-83I;ParC-80I
-	dfrA27	TEM-1D.v1^	CTX-M-15	-	SHV-28.v1^	-	OmpK35-32%	-	GyrA-83I;ParC-80I
tet(D)	dfrA27	TEM-1D.v1^	CTX-M-15	-	SHV-28.v1^	-	OmpK35-32%	-	GyrA-83I;ParC-80I
tet(A).v1	dfrA14.v2*	OXA-1;TEM-1D.v1^	CTX-M-15	KPC-3	SHV-11.v1^	35Q	-	-	-
-	dfrA14.v2*	TEM-1D.v1^	CTX-M-15	-	SHV-11.v1^	35Q	OmpK35-71%	-	-
-	-	-	-	-	-	-	-	-	-
-	-	-	-	-	-	-	-	-	-
-	-	-	-	-	-	-	-	-	-
-	-	-	-	-	SHV-108^	35Q	-	-	-
-	-	-	CTX-M-15	-	SHV-27	156D	-	-	-
-	-	-	-	-	SHV-108	35Q	-	-	-
-	-	-	-	-	SHV-26	-	-	-	-
tet(J)*	-	-	-	-	SHV-11.v1^	35Q	-	-	-
-	dfrA1.v2;dfrA27	OXA-1?;TEM-1D.v1^	CTX-M-15	-	SHV-28.v1^	-	-	-	GyrA-83F;GyrA-87A;ParC-80I
-	-	TEM-1D.v1^	-	-	SHV-27	156D	-	-	-
-	-	-	-	-	SHV-1	-	-	-	-
-	-	-	-	-	SHV-1^	-	-	-	-
-	-	-	-	-	SHV-27	156D	OmpK36-0%	-	-
-	-	-	-	-	SHV-119^	35Q	-	-	-
-	-	-	-	-	SHV-11.v1^	35Q	-	-	-
-	-	-	-	-	SHV-27	156D	-	-	-
-	-	-	-	-	SHV-42	25S	-	-	-
-	-	-	-	-	SHV-1^	-	-	-	-
-	-	-	-	-	LEN-8	-	-	-	-
-	-	-	-	-	LEN-30	-	-	-	-
-	-	-	-	KPC-3	LEN-2^	-	-	-	-
-	-	OXA-50.v2^	PDC-5^	KPC-3	LEN-2^	-	-	-	-
-	-	-	-	-	SHV-11.v1^	35Q	OmpK35-86%	-	GyrA-83Y

KLEBORATE	KLEBORATE	KLEBORATE	KLEBORATE	KLEBORATE	KLEBORATE	KLEBORATE	KLEBORATE	KLEBORATE	KLEBORATE	KLEBORATE	KLEBORATE	KLEBORATE	KLEBORATE	KLEBORATE	KLEBORATE
**truncated resistance hits**	**spurious resistance hits**	**Chr ST**	**gapA**	**infB**	**mdh**	**pgi**	**phoE**	**rpoB**	**tonB**	**ybtS**	**ybtX**	**ybtQ**	**ybtP**	**ybtA**	**irp2**
CatB4.v1?-81%	dfrA12?-73%	ST15	1	1	1	1	1	1	1	-	-	-	-	-	-
CatB4.v1?-81%	dfrA12?-73%	ST15	1	1	1	1	1	1	1	-	-	-	-	-	-
CatB4.v1?-81%	-	ST15	1	1	1	1	1	1	1	-	-	-	-	-	-
CatB4.v1?-81%	-	ST15	1	1	1	1	1	1	1	3	12	4	3	29	317
CatB4.v1?-81%	-	ST15	1	1	1	1	1	1	1	3	12	4	3	29	317
CatB4.v1?-81%	-	ST15	1	1	1	1	1	1	1	3	12	4	3	29	317
CatB4.v1?-81%	-	ST15	1	1	1	1	1	1	1	3	12	4	3	29	317
CatB4.v1?-81%	-	ST15	1	1	1	1	1	1	1	-	-	-	-	-	-
CatB4.v1?-81%	-	ST15	1	1	1	1	1	1	1	-	-	-	-	-	-
-	-	ST2623	2	83	1	1	247	1	9	-	-	-	-	-	-
-	-	ST2623	2	83	1	1	247	1	9	-	-	-	-	-	-
CatB4.v1?-81%	-	ST13	2	3	1	1	10	1	19	3	3	4	3	3	7
CatB4.v1?-81%	-	ST13	2	3	1	1	10	1	19	3	3	4	3	3	7
CatB4.v1?-81%	-	ST13	2	3	1	1	10	1	19	3	3	4	3	3	7
CatB4.v1?-81%	-	ST13	2	3	1	1	10	1	19	3	3	4	3	3	7
CatB4.v1?-81%	-	ST13	2	3	1	1	10	1	19	3	3	4	3	3	7
CatB4.v1?-81%	-	ST13	2	3	1	1	10	1	19	3	3	4	3	3	7
TEM-150*?-0%;aac(6')-Ib'.v1*?-0%	-	ST13	2	3	1	1	10	1	19	6	15	5	4	11	28
CatB4.v1?-81%	-	ST13	2	3	1	1	10	1	19	3	3	4	3	3	7*-70%
CatB4.v1?-81%	-	ST13	2	3	1	1	10	1	19	3	3	4	3	3	7
CatB4.v1?-81%	-	ST13	2	3	1	1	10	1	19	3	3	4	3	3	7
CatB4.v1?-81%	-	ST13	2	3	1	1	10	1	19	3	3	4	3	3	7
CatB4.v1?-81%	-	ST13	2	3	1	1	10	1	19	3	3	4	3	3	7
CatB4.v1?-81%	-	ST13	2	3	1	1	10	1	19	3	3	4	3	3	7
-	-	ST35	2	1	2	1	10	1	19	10	8	10	7	18	4
dfrA14.v2*?-0%	SHV-187*?-42%	ST35	2	1	2	1	10	1	19	10	8*	10	7	18	4
CatB4.v1?-81%	fosA7*?-0%	ST405	2	1	62	3	10	4	110	6	62	60	4	1	145
CatB4.v1?-81%	fosA7*?-0%	ST405	2	1	62	3	10	4	110	6	62	60	4	1	145
CatB4.v1?-81%	-	ST20	2	3	1	1	4	4	4	-	-	-	-	-	-
CatB4.v1?-81%	-	ST20	2	3	1	1	4	4	4	-	-	-	-	-	-
CatB4.v1?-81%	-	ST20	2	3	1	1	4	4	4	-	-	-	-	-	-
CatB4.v1?-81%	-	ST20	2	3	1	1	4	4	4	-	-	-	-	-	-
CatB4.v1?-81%	-	ST20	2	3	1	1	4	4	4	-	-	-	-	-	-
-	-	ST147	3	4	6	1	7	4	38	6	15	5	4	11	33
-	-	ST147	3	4	6	1	7	4	38	6	15	5	4*	11	33*
-	-	ST147	3	4	6	1	7	4	38	6	15	5	4*	11	33*
-	-	ST147	3	4	6	1	7	4	38	6*	15	5	4	11	33
-	-	ST147	3	4	6	1	7	4	38	6	15	5	4*	11	33*
-	-	ST147	3	4	6	1	7	4	38	6	15	5	4*	11	33*
-	-	ST147	3	4	6	1	7	4	38	-	-	-	-	-	-
CatB4.v1?-81%;SHV-187*-78%;tet(A).v1?-0%	-	ST29	2	3	2	2	6	4	4	4	4	5	4	1	69
SHV-187*-78%;tet(A).v1?-0%	-	ST29	2	3	2	2	6	4	4	4	4	5	4	1	69
SHV-187*-78%;tet(A).v1?-0%	-	ST29	2	3	2	2	6	4	4	4	4	5	4	1	69
CatB4.v1?-81%;SHV-187*-78%;tet(A).v1?-0%	-	ST29	2	3	2	2	6	4	4	4	4	5	4	1	69
CatB4.v1?-81%;SHV-187*-78%;tet(A).v1?-0%	-	ST29	2	3	2	2	6	4	4	4	4	5	4	1	69
CatB4.v1?-81%;SHV-187*-78%;tet(A).v1?-0%	-	ST29	2	3	2	2	6	4	4	4	4	5	4	1	69
CatB4.v1?-81%;SHV-187*-78%;tet(A).v1?-0%	-	ST29	2	3	2	2	6	4	4	4	4	5	4	1	69
-	-	ST37	2	9	2	1	13	1	16	6	15	22	4	1	37
-	-	ST37	2	9	2	1	13	1	16	6	15	22	4	1	37
-	-	ST45	2	1	1	6	7	1	12	3	12	4	11	3	21
-	CatB4.v1?-46%;SHV-187*?-47%	ST45	2	1	1	6	7	1	12	-	-	-	-	-	-
-	-	ST45	2	1	1	6	7	1	12	3	12	4	11	3	21
-	SHV-187*?-47%	ST45	2	1	1	6	7	1	12	-	-	-	-	-	-
-	-	ST45	2	1	1	6	7	1	12	3	12	4	11	3	21*
-	SHV-187*?-47%	ST45	2	1	1	6	7	1	12	3	12	4	11	3	21*
-	-	ST45	2	1	1	6	7	1	12	3	12	4	11	3	21*
-	SHV-187*?-47%	ST45	2	1	1	6	7	1	12	3	12	4	11	3	21*
-	SHV-161?-0%;SHV-187*?-47%	ST45	2	1	1	6	7	1	12	3	12	4	11	3	21*
-	SHV-187*?-47%	ST45	2	1	1	6	7	1	12	3	12	4	11	3	21*
-	SHV-187*?-47%	ST45	2	1	1	6	7	1	12	3	12	4	11	3	21*
-	SHV-187*?-47%	ST45	2	1	1	6	7	1	12	3	12	4	11	3	21*
-	SHV-187*?-47%	ST45	2	1	1	6	7	1	12	3	12	4	11	3	21*
-	SHV-187*?-47%	ST45	2	1	1	6	7	1	12	3	12	4	11	3	21*
-	SHV-187*?-47%	ST45	2	1	1	6	7	1	12	3	12	4	11	3	21*
-	SHV-187*?-47%	ST45	2	1	1	6	7	1	12	3	12	4	11	3	21*
-	SHV-187*?-47%	ST45	2	1	1	6	7	1	12	3	12	4	11	3	21*
-	SHV-187*?-47%	ST45	2	1	1	6	7	1	12	3	12	4	11	3	21*
-	-	ST45	2	1	1	6	7	1	12	3	12	4	11	3	21*
-	SHV-187*?-47%	ST45	2	1	1	6	7	1	12	3	12	4	11	3	21*
-	SHV-187*?-47%	ST45	2	1	1	6	7	1	12	3	12	4	11	3	21*
-	-	ST45	2	1	1	6	7	1	12	3	12	4	11	3	21*
-	-	ST45	2	1	1	6	7	1	12	3	12	4	11	3	21*
-	-	ST45	2	1	1	6	7	1	12	3	12	4	11	3	21*
-	SHV-187*?-47%	ST45	2	1	1	6	7	1	12	3	12	4	11	3	21*
-	SHV-187*?-47%	ST45	2	1	1	6	7	1	12	3	12	4	11	3	21*
SHV-102*?-84%	-	ST45	2	1	1	6	7	1	12	3	12	4	11	3	21*
-	-	ST45	2	1	1	6	7	1	12	3	12	4	11	3	21*
-	SHV-187*?-47%	ST45	2	1	1	6	7	1	12	3	12	4	11	3	21*
-	SHV-187*?-47%	ST45	2	1	1	6	7	1	12	3	12	4	11	3	21*
-	SHV-187*?-47%	ST45	2	1	1	6	7	1	12	3	12	4	11	3	21*
-	SHV-187*?-47%	ST45	2	1	1	6	7	1	12	3	12	4	11	3	21*
-	-	ST45	2	1	1	6	7	1	12	3	12	4	11	3	21*
-	SHV-187*?-47%	ST45	2	1	1	6	7	1	12	3	12	4	11	3	21*
-	SHV-145?-0%;SHV-187*?-47%	ST45	2	1	1	6	7	1	12	3	12	4	11	3	21*
-	SHV-187*?-47%	ST45	2	1	1	6	7	1	12	3	12	4	11	3	21*
-	SHV-187*?-47%	ST45	2	1	1	6	7	1	12	3	12	4	11	3	21*
-	-	ST307	4	1	2	52	1	1	7	-	-	-	-	-	-
-	-	ST307	4	1	2	52	1	1	7	-	-	-	-	-	-
-	-	ST307	4	1	2	52	1	1	7	-	-	-	-	-	-
-	-	ST307	4	1	2	52	1	1	7	-	-	-	-	-	-
-	-	ST307	4	1	2	52	1	1	7	-	-	-	-	-	-
CatB4.v1?-81%	-	ST348	2	1	20	1	12	15	16	86	12	117	3	1	274
-	-	ST348	2	1	20	1	12	15	16	86	12	117	3	1	274
-	-	NA	-	-	-	-	-	-	-	-	-	-	-	-	-
-	-	NA	-	-	-	-	-	-	-	-	-	-	-	-	-
-	-	NA	-	-	-	-	-	-	-	-	-	-	-	-	-
-	-	ST7	3	1	1	4	3	1	34	5	11	6*	5	1	25
-	TEM-104?-67%	ST4742	4	1	2	36	12	1	14	75	34	37	44	47	104*
-	-	ST33	2	3	5	1	12	4	9	-	-	-	-	-	-
-	-	ST643	2	3	6	75	9	7	137	-	-	-	-	-	-
-	-	ST111	2	1	5	1	17	4	42	-	-	-	-	-	-
CatB4.v1?-81%	-	ST15	1	1	1	1	1	1	1	3	12	4*	3	29	317*
-	-	ST278	4	1	1	1	12	1	4	-	-	-	-	-	-
-	-	ST1452	2	5	6	4	64	2	12	-	-	-	-	-	-
-	-	ST461	3	1	13	34	63	1	64	-	-	-	-	-	-
-	-	ST1805	4	3	1	1	9	27	14	-	-	-	-	-	-
-	-	ST160	10	7	1	1	1	1	35	18	20	27	18	13	81
-	-	ST17-1LV	2	1	1	1	4	4	4*	-	-	-	-	-	-
-	-	ST239	4	1	5	1	9	11	24	-	-	-	-	-	-
-	-	ST540	2	1	71	1	3	3	18	-	-	-	-	-	-
-	-	ST152	2	3	2	1	1	4	56	-	-	-	-	-	-
-	-	ST318-1LV	16	24	21	31	41	22	67	-	-	-	-	-	-
-	-	ST360	16	24	21	53	47	17	67	-	-	-	-	-	-
-	-	ST1562	16	24	36	27	29	22	105	-	-	-	-	-	-
-	-	ST1562	16	24	36	27	29	22	105	-	-	-	-	-	-
-	-	ST38	2	1	2	1	2	2	2	-	-	-	-	-	-

KLEBORAT	KLEBORAT	KLEBORAT	KLEBORATE	KLEBORAT	KLEBORAT	KLEBORAT	KLEBORAT	KLEBORAT	KLEBORAT	KLEBORAT	KLEBORAT	KLEBORAT	KLEBORAT	KLEBORAT	KLEBORAT	KLEBORAT	KLEBORAT	KLEBORAT	KLEBORAT	KLEBORAT
**irp1**	**ybtU**	**ybtT**	**ybtE**	**fyuA**	**clbA**	**clbB**	**clbC**	**clbD**	**clbE**	**clbF**	**clbG**	**clbH**	**clbI**	**clbL**	**clbM**	**clbN**	**clbO**	**clbP**	**clbQ**	**iucA**
-	-	-	-	-	-	-	-	-	-	-	-	-	-	-	-	-	-	-	-	-
-	-	-	-	-	-	-	-	-	-	-	-	-	-	-	-	-	-	-	-	-
-	-	-	-	-	-	-	-	-	-	-	-	-	-	-	-	-	-	-	-	-
360	3	80	9	2	-	-	-	-	-	-	-	-	-	-	-	-	-	-	-	-
360	3	80	9	2	-	-	-	-	-	-	-	-	-	-	-	-	-	-	-	-
360	3	80	9	2	-	-	-	-	-	-	-	-	-	-	-	-	-	-	-	-
360	3	80	9	2	-	-	-	-	-	-	-	-	-	-	-	-	-	-	-	-
-	-	-	-	-	-	-	-	-	-	-	-	-	-	-	-	-	-	-	-	-
-	-	-	-	-	-	-	-	-	-	-	-	-	-	-	-	-	-	-	-	-
-	-	-	-	-	-	-	-	-	-	-	-	-	-	-	-	-	-	-	-	-
-	-	-	-	-	-	-	-	-	-	-	-	-	-	-	-	-	-	-	-	-
7	3	3	3	32	-	-	-	-	-	-	-	-	-	-	-	-	-	-	-	-
7	3	3	3	32	-	-	-	-	-	-	-	-	-	-	-	-	-	-	-	-
7	3	3	3	32	-	-	-	-	-	-	-	-	-	-	-	-	-	-	-	-
7	3	3	3	32	-	-	-	-	-	-	-	-	-	-	-	-	-	-	-	-
7	3	3	3	32	-	-	-	-	-	-	-	-	-	-	-	-	-	-	-	-
7	3	3	3	32	-	-	-	-	-	-	-	-	-	-	-	-	-	-	-	-
26	2	11	4	2	2	53	2	2	2	2	2	8	2	2	2	2	2	2	2	-
7	3	3	3	32	-	-	-	-	-	-	-	-	-	-	-	-	-	-	-	-
7	3	3	3	32	-	-	-	-	-	-	-	-	-	-	-	-	-	-	-	-
7	3	3	3	32	-	-	-	-	-	-	-	-	-	-	-	-	-	-	-	-
7	3	3	3	32	-	-	-	-	-	-	-	-	-	-	-	-	-	-	-	-
7	3	3	3	32	-	-	-	-	-	-	-	-	-	-	-	-	-	-	-	-
7	3	3	3	32	-	-	-	-	-	-	-	-	-	-	-	-	-	-	-	-
288*	7	8	5	37	-	-	-	-	-	-	-	-	-	-	-	-	-	-	-	-
288	7	8	5	37	-	-	-	-	-	-	-	-	-	-	-	-	-	-	-	-
148	2	39	69	2	-	-	-	-	-	-	-	-	-	-	-	-	-	-	-	-
148	2	39	69	2	-	-	-	-	-	-	-	-	-	-	-	-	-	-	-	-
-	-	-	-	-	-	-	-	-	-	-	-	-	-	-	-	-	-	-	-	-
-	-	-	-	-	-	-	-	-	-	-	-	-	-	-	-	-	-	-	-	-
-	-	-	-	-	-	-	-	-	-	-	-	-	-	-	-	-	-	-	-	-
-	-	-	-	-	-	-	-	-	-	-	-	-	-	-	-	-	-	-	-	-
-	-	-	-	-	-	-	-	-	-	-	-	-	-	-	-	-	-	-	-	-
23*	2	11	4	2	-	-	-	-	-	-	-	-	-	-	-	-	-	-	-	-
23*	2	11	4	2	-	-	-	-	-	-	-	-	-	-	-	-	-	-	-	-
23*	2	11	4	2	-	-	-	-	-	-	-	-	-	-	-	-	-	-	-	-
23*	2	11	4	2	-	-	-	-	-	-	-	-	-	-	-	-	-	-	-	-
23*	2	11	4	2	-	-	-	-	-	-	-	-	-	-	-	-	-	-	-	-
23*	2	11	4	2	-	-	-	-	-	-	-	-	-	-	-	-	-	-	-	-
-	-	-	-	-	-	-	-	-	-	-	-	-	-	-	-	-	-	-	-	-
62	2	4	4	20	-	-	-	-	-	-	-	-	-	-	-	-	-	-	-	-
62	2	4	4	20	-	-	-	-	-	-	-	-	-	-	-	-	-	-	-	-
62	2	4	4	20	-	-	-	-	-	-	-	-	-	-	-	-	-	-	-	-
62	2	4	4	20	-	-	-	-	-	-	-	-	-	-	-	-	-	-	-	-
62	2	4	4	20	-	-	-	-	-	-	-	-	-	-	-	-	-	-	-	-
62	2	4	4	20	-	-	-	-	-	-	-	-	-	-	-	-	-	-	-	-
62	2	4	4	20	-	-	-	-	-	-	-	-	-	-	-	-	-	-	-	-
44	14	1	4	17	-	-	-	-	-	-	-	-	-	-	-	-	-	-	-	-
44	14	1	4	17	-	-	-	-	-	-	-	-	-	-	-	-	-	-	-	-
18	3	10	16	2	-	-	-	-	-	-	-	-	-	-	-	-	-	-	-	-
-	-	-	-	-	-	-	-	-	-	-	-	-	-	-	-	-	-	-	-	-
18	3	10	16	2	-	-	-	-	-	-	-	-	-	-	-	-	-	-	-	-
-	-	-	-	-	-	-	-	-	-	-	-	-	-	-	-	-	-	-	-	-
18	3	10	16	2	-	-	-	-	-	-	-	-	-	-	-	-	-	-	-	-
18	3	10	16	2	-	-	-	-	-	-	-	-	-	-	-	-	-	-	-	-
18	3	10	16	2	-	-	-	-	-	-	-	-	-	-	-	-	-	-	-	-
18	3	10	16	2	-	-	-	-	-	-	-	-	-	-	-	-	-	-	-	-
18	3	10	16	2	-	-	-	-	-	-	-	-	-	-	-	-	-	-	-	-
18	3	10	16	2	-	-	-	-	-	-	-	-	-	-	-	-	-	-	-	-
18	3	10	16	2	-	-	-	-	-	-	-	-	-	-	-	-	-	-	-	-
18	3	10	16	2	-	-	-	-	-	-	-	-	-	-	-	-	-	-	-	-
18	3	10	16	2	-	-	-	-	-	-	-	-	-	-	-	-	-	-	-	-
18	3	10	16	2	-	-	-	-	-	-	-	-	-	-	-	-	-	-	-	-
18	3	10	16	2	-	-	-	-	-	-	-	-	-	-	-	-	-	-	-	-
18	3	10	16	2	-	-	-	-	-	-	-	-	-	-	-	-	-	-	-	-
18	3	10	16	2	-	-	-	-	-	-	-	-	-	-	-	-	-	-	-	-
18	3	10	16	2	-	-	-	-	-	-	-	-	-	-	-	-	-	-	-	-
18	3	10	16	2	-	-	-	-	-	-	-	-	-	-	-	-	-	-	-	-
18	3	10	16	2	-	-	-	-	-	-	-	-	-	-	-	-	-	-	-	-
18	3	10	16	2	-	-	-	-	-	-	-	-	-	-	-	-	-	-	-	-
18	3	10	16	2	-	-	-	-	-	-	-	-	-	-	-	-	-	-	-	-
18	3	10	16	2	-	-	-	-	-	-	-	-	-	-	-	-	-	-	-	-
18	3	10	16	2	-	-	-	-	-	-	-	-	-	-	-	-	-	-	-	-
18	3	10	16	2	-	-	-	-	-	-	-	-	-	-	-	-	-	-	-	-
18	3	10	16	2	-	-	-	-	-	-	-	-	-	-	-	-	-	-	-	-
18	3	10	16	2	-	-	-	-	-	-	-	-	-	-	-	-	-	-	-	-
18	3	10	16	2	-	-	-	-	-	-	-	-	-	-	-	-	-	-	-	-
18	3	10	16	2	-	-	-	-	-	-	-	-	-	-	-	-	-	-	-	-
18	3	10	16	2	-	-	-	-	-	-	-	-	-	-	-	-	-	-	-	-
18	3	10	16	2	-	-	-	-	-	-	-	-	-	-	-	-	-	-	-	-
18	3	10	16	2	-	-	-	-	-	-	-	-	-	-	-	-	-	-	-	-
18	3	10	16	2	-	-	-	-	-	-	-	-	-	-	-	-	-	-	-	-
18	3	10	16	2	-	-	-	-	-	-	-	-	-	-	-	-	-	-	-	-
18	3	10	16	2	-	-	-	-	-	-	-	-	-	-	-	-	-	-	-	-
-	-	-	-	-	-	-	-	-	-	-	-	-	-	-	-	-	-	-	-	-
-	-	-	-	-	-	-	-	-	-	-	-	-	-	-	-	-	-	-	-	-
-	-	-	-	-	-	-	-	-	-	-	-	-	-	-	-	-	-	-	-	-
-	-	-	-	-	-	-	-	-	-	-	-	-	-	-	-	-	-	-	-	-
-	-	-	-	-	-	-	-	-	-	-	-	-	-	-	-	-	-	-	-	-
411	70	88	4	153	-	-	-	-	-	-	-	-	-	-	-	-	-	-	-	-
411*	70	88	4	153	-	-	-	-	-	-	-	-	-	-	-	-	-	-	-	-
-	-	-	-	-	-	-	-	-	-	-	-	-	-	-	-	-	-	-	-	-
-	-	-	-	-	-	-	-	-	-	-	-	-	-	-	-	-	-	-	-	-
-	-	-	-	-	-	-	-	-	-	-	-	-	-	-	-	-	-	-	-	-
37	9	5	5	11*	-	-	-	-	-	-	-	-	-	-	-	-	-	-	-	-
227*	25	23	45	118	-	-	-	-	-	-	-	-	-	-	-	-	-	-	-	-
-	-	-	-	-	-	-	-	-	-	-	-	-	-	-	-	-	-	-	-	-
-	-	-	-	-	-	-	-	-	-	-	-	-	-	-	-	-	-	-	-	-
-	-	-	-	-	-	-	-	-	-	-	-	-	-	-	-	-	-	-	-	-
360	3	80	9	2	-	-	-	-	-	-	-	-	-	-	-	-	-	-	-	-
-	-	-	-	-	-	-	-	-	-	-	-	-	-	-	-	-	-	-	-	38
-	-	-	-	-	-	-	-	-	-	-	-	-	-	-	-	-	-	-	-	-
-	-	-	-	-	-	-	-	-	-	-	-	-	-	-	-	-	-	-	-	-
-	-	-	-	-	-	-	-	-	-	-	-	-	-	-	-	-	-	-	-	-
38	9	8	26	35	-	-	-	-	-	-	-	-	-	-	-	-	-	-	-	-
-	-	-	-	-	-	-	-	-	-	-	-	-	-	-	-	-	-	-	-	-
-	-	-	-	-	-	-	-	-	-	-	-	-	-	-	-	-	-	-	-	-
-	-	-	-	-	-	-	-	-	-	-	-	-	-	-	-	-	-	-	-	-
-	-	-	-	-	-	-	-	-	-	-	-	-	-	-	-	-	-	-	-	-
-	-	-	-	-	-	-	-	-	-	-	-	-	-	-	-	-	-	-	-	-
-	-	-	-	-	-	-	-	-	-	-	-	-	-	-	-	-	-	-	-	-
-	-	-	-	-	-	-	-	-	-	-	-	-	-	-	-	-	-	-	-	-
-	-	-	-	-	-	-	-	-	-	-	-	-	-	-	-	-	-	-	-	-
-	-	-	-	-	-	-	-	-	-	-	-	-	-	-	-	-	-	-	-	-

KLEBORATE	KLEBORATE	KLEBORATE	KLEBORATE	KLEBORATE	KLEBORATE	KLEBORATE	KLEBORATE
**iucB**	**iucC**	**iucD**	**iutA**	**iroB**	**iroC**	**iroD**	**iroN**
-	-	-	-	-	-	-	-
-	-	-	-	-	-	-	-
-	-	-	-	-	-	-	-
-	-	-	-	-	-	-	-
-	-	-	-	-	-	-	-
-	-	-	-	-	-	-	-
-	-	-	-	-	-	-	-
-	-	-	-	-	-	-	-
-	-	-	-	-	-	-	-
-	-	-	-	-	-	-	-
-	-	-	-	-	-	-	-
-	-	-	-	-	-	-	-
-	-	-	-	-	-	-	-
-	-	-	-	-	-	-	-
-	-	-	-	-	-	-	-
-	-	-	-	-	-	-	-
-	-	-	-	-	-	-	-
-	-	-	-	-	-	-	-
-	-	-	-	-	-	-	-
-	-	-	-	-	-	-	-
-	-	-	-	-	-	-	-
-	-	-	-	-	-	-	-
-	-	-	-	-	-	-	-
-	-	-	-	-	-	-	-
-	-	-	-	-	-	-	-
-	-	-	-	-	-	-	-
-	-	-	-	-	-	-	-
-	-	-	-	-	-	-	-
-	-	-	-	-	-	-	-
-	-	-	-	-	-	-	-
-	-	-	-	-	-	-	-
-	-	-	-	-	-	-	-
-	-	-	-	-	-	-	-
-	-	-	-	-	-	-	-
-	-	-	-	-	-	-	-
-	-	-	-	-	-	-	-
-	-	-	-	-	-	-	-
-	-	-	-	-	-	-	-
-	-	-	-	-	-	-	-
-	-	-	-	-	-	-	-
-	-	-	-	-	-	-	-
-	-	-	-	-	-	-	-
-	-	-	-	-	-	-	-
-	-	-	-	-	-	-	-
-	-	-	-	-	-	-	-
-	-	-	-	-	-	-	-
-	-	-	-	-	-	-	-
-	-	-	-	-	-	-	-
-	-	-	-	-	-	-	-
-	-	-	-	-	-	-	-
-	-	-	-	-	-	-	-
-	-	-	-	-	-	-	-
-	-	-	-	-	-	-	-
-	-	-	-	-	-	-	-
-	-	-	-	-	-	-	-
-	-	-	-	-	-	-	-
-	-	-	-	-	-	-	-
-	-	-	-	-	-	-	-
-	-	-	-	-	-	-	-
-	-	-	-	-	-	-	-
-	-	-	-	-	-	-	-
-	-	-	-	-	-	-	-
-	-	-	-	-	-	-	-
-	-	-	-	-	-	-	-
-	-	-	-	-	-	-	-
-	-	-	-	-	-	-	-
-	-	-	-	-	-	-	-
-	-	-	-	-	-	-	-
-	-	-	-	-	-	-	-
-	-	-	-	-	-	-	-
-	-	-	-	-	-	-	-
-	-	-	-	-	-	-	-
-	-	-	-	-	-	-	-
-	-	-	-	-	-	-	-
-	-	-	-	-	-	-	-
-	-	-	-	-	-	-	-
-	-	-	-	-	-	-	-
-	-	-	-	-	-	-	-
-	-	-	-	-	-	-	-
-	-	-	-	-	-	-	-
-	-	-	-	-	-	-	-
-	-	-	-	-	-	-	-
-	-	-	-	-	-	-	-
-	-	-	-	-	-	-	-
-	-	-	-	-	-	-	-
-	-	-	-	-	-	-	-
-	-	-	-	-	-	-	-
-	-	-	-	-	-	-	-
-	-	-	-	-	-	-	-
-	-	-	-	-	-	-	-
-	-	-	-	-	-	-	-
-	-	-	-	-	-	-	-
-	-	-	-	-	-	-	-
-	-	-	-	-	-	-	-
-	-	-	-	-	-	-	-
-	-	-	-	25*	33*-0%	-	15*
-	-	-	-	25*	33*-0%	-	15*
-	-	-	-	25*	33*-0%	-	15*
-	-	-	-	-	-	-	-
-	-	-	-	-	-	-	-
-	-	-	-	-	-	-	-
-	-	-	-	-	-	-	-
-	-	-	-	-	-	-	-
-	-	-	-	-	-	-	-
12	33	35	61	23	48	17	23
-	-	-	-	-	-	-	-
-	-	-	-	-	-	-	-
-	-	-	-	-	-	-	-
-	-	-	-	-	-	-	-
-	-	-	-	-	-	-	-
-	-	-	-	-	-	-	-
-	-	-	-	-	-	-	-
-	-	-	-	-	-	-	-
-	-	-	-	-	-	-	-
-	-	-	-	-	-	-	-
-	-	-	-	-	-	-	-
-	-	-	-	-	-	-	-
-	-	-	-	-	-	-	-

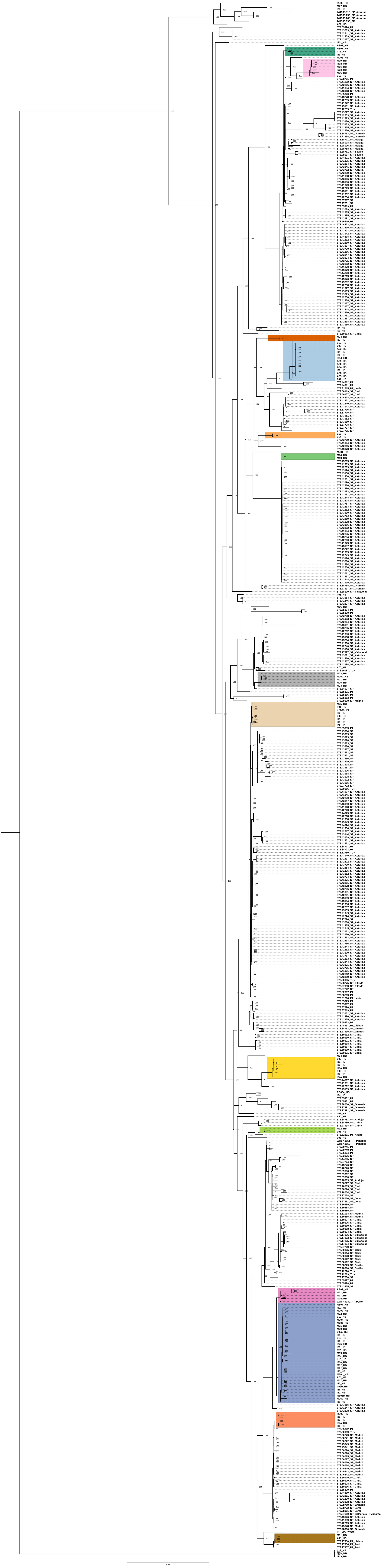

Supplement: Supplementary file 1 [file biology-14-01795-s001.zip › biology-3996026-supplementary.pdf]
